# Comparative study of functional training and traditional resistance training on lower-limb strength performance in male adolescent volleyball players: a randomized controlled trial

**DOI:** 10.3389/fphys.2025.1629055

**Published:** 2025-09-02

**Authors:** Yuan Gao, Yale Yang, Chenyang Xian, Zetao Wang

**Affiliations:** ^1^ China West Normal University, Nanchong, China; ^2^ Shandong swimming Management Center, Nanchong, China; ^3^ Sichuan Winshare Vocational College, Nanchong, China; ^4^ Beijing Zhongguancun High School Zhichun Branch Camps, Nanchong, China

**Keywords:** functional training, traditional resistance training, adolescent athletes, lower-limb strength, randomized controlled trial, volleyball

## Abstract

**Background:**

This study aimed to compare the effects of a functional strength-training program designed around volleyball-specific movements with those of traditional resistance training on lower-limb performance in male adolescent volleyball players.

**Methods:**

In a randomized controlled trial, 52 male volleyball athletes (age 13 ± 2 years; ≥2 years of formal volleyball training) from a sports school in Huangshi, Hubei Province, China, were allocated 1:1 to a functional training group (n = 26) or a traditional resistance training group (n = 26). The intervention lasted 12 weeks, with three 90-min sessions per week. Pre- and post-training assessments of lower-limb strength and overall fitness included six measures: standing reach height, approach reach height, half-meter square agility test, standing long jump, 30 m sprint, and 1,500 m run. Data were analyzed in SPSS version 26.0 using paired-samples and independent-samples t-tests, with α = 0.05.

**Results:**

In the functional training group, standing reach height increased from 2.81 ± 0.10 m to 2.88 ± 0.09 m (P = 0.025), and approach reach height from 2.87 ± 0.12 m to 2.95 ± 0.11 m (P = 0.034). The half-meter square agility time decreased from 18.09 ± 0.98 s to 17.50 ± 1.06 s but did not reach statistical significance (P = 0.075). In the traditional resistance training group, only the half-meter square agility test showed a significant improvement (P = 0.028), with no significant changes in the other five measures (all P > 0.05). Between-group comparisons revealed no significant differences across any outcome (all P > 0.05).

**Conclusion:**

Functional training appears superior to traditional resistance training for enhancing lower-limb explosive power in adolescent volleyball players, whereas traditional resistance training retains an advantage in agility. We recommend integrating both functional and traditional strength modalities in youth volleyball conditioning to foster comprehensive development of lower-limb strength and sport-specific performance.

## 1 Introduction

In recent years, as training concepts have continually evolved, functional training has gradually become an important means of improving the physical fitness level and sport-specific capabilities of adolescent athletes (Crawford et al., 2018; Pereira et al., 2025). Functional training is a training approach that emphasizes movement patterns, core stability, balance, and multi-joint synergistic force production, distinguishing it from the traditional resistance training model that focuses on single muscle groups and fixed trajectories. This method is oriented towards functional movement, more closely reflecting the actual actions required in sport, and is thus considered to have unique advantages in enhancing sport-specific performance (Wan et al., 2025; Weiss et al., 2010; Xiao et al., 2025; 2021; Yildiz et al., 2019). Existing research indicates that functional training can effectively improve key fitness indicators in adolescent athletes—such as strength (Oliver and Di Brezzo, 2009), speed (Tomljanović et al., 2011), agility, and endurance (Oliver and Di Brezzo, 2009) and can further enhance athletic performance (Abood et al., 2022; Alhenawy, 2022; Zuo et al., 2022).

Volleyball is a ball sport that highly depends on lower-limb explosive power, rapid movement ability, and reactive agility (Bobula et al., 2024; Tramel et al., 2019). Whether in the high-intensity vertical jumps needed for spiking and blocking, or in the frequent lateral movements and changes of direction during defense, the level of lower-limb muscular strength directly relates to an athlete’s on-court performance (Deng et al., 2025; George Lockie, 2019; Tramel et al., 2019). Performance measures such as vertical jump height, approach jump height, 30 m sprint time, and standing long jump distance have been widely used to assess the lower-limb strength and explosive power of volleyball players (Bobula et al., 2024). For adolescent volleyball athletes in the pubertal stage (13 ± 2 years old), determining how to effectively enhance sport-specific fitness while ensuring training safety is an urgent issue in youth competitive sports training.

Currently, traditional lower-limb resistance training (e.g., barbell squats, deadlifts) remains widely applied in youth training (Behm et al., 2008; Faigenbaum and Myer, 2010). While these methods aim to increase maximal muscle strength and can indeed strengthen muscle groups (Barbalho et al., 2018; Lloyd et al., 2016; Lopez et al., 2021), there may be limitations in translating those gains to sport performance due to differences between training movements and actual competition actions (Suarez et al., 2019). In contrast, functional training emphasizes dynamic, multi-directional, multi-joint participation, which better aligns with the complex and variable demands of volleyball competition and may more effectively improve athletes’ postural control, technical consistency, and overall physical performance (Fernandez-Fernandez et al., 2020; Xiao et al., 2021). However, most existing comparative studies on the effects of functional *versus* traditional training have focused on adults or other sports (Abood et al., 2022; Alonso-Fernández et al., 2017; Baron et al., 2019; Chen and Chen, 2023; Fernandez-Fernandez et al., 2020; Kovac et al., 2022; Lee et al., 2023; Łysoń-Uklańska et al., 2021), and empirical randomized controlled trials specifically targeting adolescent volleyball players remain scarce (Wan et al., 2025). It is worth noting that adolescence represents a critical period of rapid neuromuscular adaptation (Aoki et al., 2017; Tumkur Anil Kumar et al., 2021), during which scientifically designed training interventions may have a profound impact on athletic performance (Granacher et al., 2018; Tumkur Anil Kumar et al., 2021). Therefore, it is necessary to systematically investigate the real-world effects of these two training modalities in this population.

This study aims to compare the effects of a functional training program designed around volleyball-specific movements with those of traditional resistance training on lower-limb strength performance in male adolescent volleyball players. Using a randomized controlled trial design, subjects were assigned to either a functional training group or a traditional training group for periodic intervention, with multiple fitness indicators—such as standing reach, approach reach, and standing long jump—used for evaluation. The results of this study are intended to provide theoretical and practical guidance for the scientific training of youth volleyball players and to offer empirical support for the application of functional training in youth sport-specific conditioning.

## 2 Study participants and methods

### 2.1 Participants

The sample size for this study was estimated *a priori* using G*Power 3.1 software. The analysis selected was under “t tests” for “Means: Difference between two independent means (two groups).” With parameters set as an effect size of 0.8, a significance level (α) of 0.05, and a statistical power of 0.8, the software estimated that a total of 52 participants (26 per group) were required. However, considering practical difficulties in participant recruitment, an adjusted calculation was performed in G*Power. By maintaining the effect size at 0.8 and limiting the maximum feasible number of participants per group to 20, the recalculated statistical power was 0.69. Based on this adjustment, the study recruited 40 participants (20 per group) to meet the basic statistical requirements. To address potential attrition during the intervention period, approximately 23% additional participants were planned in the initial sample size estimation. The target of 52 participants was set to ensure that the final number of participants would still meet the required statistical power.

**FIGURE 1:**
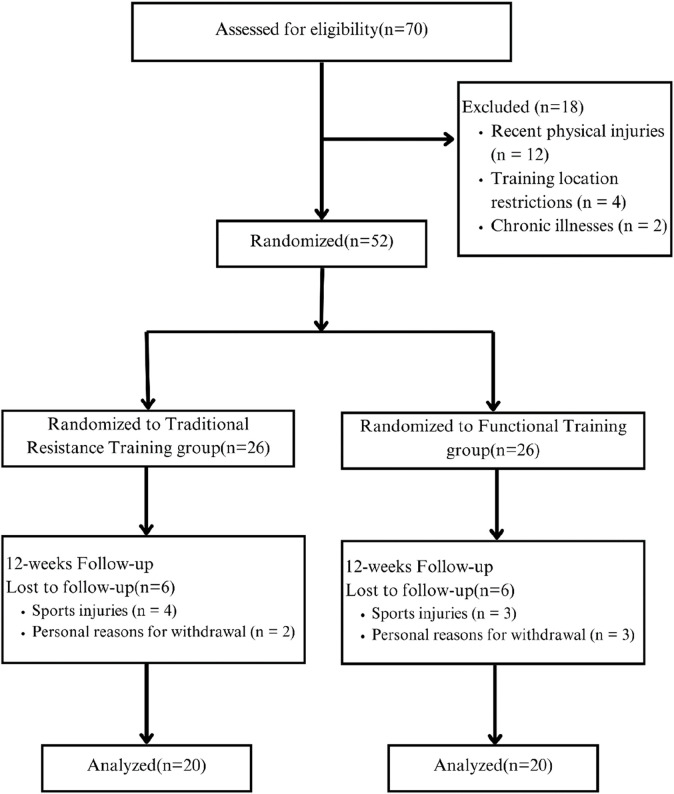
Participant flow chart.

A total of 70 male adolescent athletes were initially screened from a sports school in Huangshi, Hubei Province, based on the inclusion criteria: age (13 ± 2 years) and a minimum of 2 years of formal volleyball training experience, which included participation in structured team training, skill development, and involvement in more than two official competitions. According to the exclusion criteria—recent injury (n = 12), inaccessible training location (n = 4), or chronic illness (n = 2)—18 participants were excluded. The remaining 52 participants completed baseline measurements and were randomly allocated, using computer-generated random numbers, in a 1:1 ratio, to a functional training group (n = 26) or a traditional training group (n = 26). During the 12-week intervention period, 12 participants withdrew for personal reasons unrelated to the training intervention, such as injury or scheduling conflicts, with six dropouts in each group. Consequently, 40 participants (20 per group) completed the full protocol and were included in the per-protocol analysis (Figure 1). This study was approved by the Ethics Committee of China West Normal University, Hubei Province (Ethics No.: 2025LLSC0062). All participants and their legal guardians provided written informed consent after being fully informed of the study procedures and potential risks prior to the commencement of the trial.

### 2.2 Study design

This study employed a randomized grouping design. The 40 participants were allocated by simple random draw into an experimental group and a control group (20 per group). To minimize intervention bias, an assessor-blinded design was adopted—that is, the testers remained unaware of each subject’s group assignment throughout the trial. In addition, all assessors underwent systematic pre-test training and participated in consistency training or reliability testing to ensure uniform assessment procedures. The control group performed a traditional lower-limb strength-training protocol, while the experimental group received a functional strength-training intervention.

Prior to intervention, all participants underwent standardized baseline measurements and lower-limb performance tests at the men’s volleyball training facility in Huangshi, Hubei Province. These assessments included age, height, body mass, and sport-specific explosive-power tests (e.g., standing reach, approach reach, standing long jump).

The procedure for determining lower-limb one-repetition maximum (1-RM) was as follows:

1. Warm-up Sets

Set 1: 50% of the estimated 1-RM for 5–10 repetitions, rest 1 min.

Set 2: 70%–75% of the estimated 1-RM for three to five repetitions, rest 1 min.

Set 3: 85%–90% of the estimated 1-RM for two to three repetitions, rest 3 min.

2. Formal 1-RM testing

Initial test load was based on the warm-up feedback.

If one full, standard repetition was successfully completed, the load was increased by 2.5 kg; if the attempt failed, the load was decreased by 2.5 kg.

Rest intervals of 2–4 min were taken between attempts.

Testing continued for 3–5 rounds until the maximum load permitting a standard squat was identified as the subject’s 1-RM.

Baseline characteristics for the two groups are shown in Table 1. In the control group, age was 13.62 ± 1.23 years, height 176.77 ± 7.19 cm, and body mass 70.62 ± 8.64 kg; in the experimental group, age was 13.43 ± 1.09 years, height 175.50 ± 4.90 cm, and body mass 65.18 ± 7.58 kg. Independent-samples t-tests in SPSS revealed no significant differences between groups in age (P = 0.613 > 0.05), height (P = 0.518 > 0.05), or body mass (P = 0.109 > 0.05), indicating comparability at baseline. Although the P value for body mass approached significance (0.109), it did not meet the threshold (P < 0.05), and thus is not expected to materially affect subsequent outcomes. Overall, the two groups were well matched in basic physiological characteristics, providing a reliable foundation for comparing intervention effects.

**TABLE 1:** Participant baseline characteristics.

Basic information	Control group (n = 26)	Experimental group (n = 26)	P-value
Age (years)	13.62 ± 1.26	13.43 ± 1.09	0.613
Height (cm)	176.77 ± 7.19	175.50 ± 4.90	0.518
Weight (kg)	70.62 ± 8.64	65.18 ± 7.58	0.109
Standing Reach Height (m)	2.85 ± 0.13	2.81 ± 0.10	0.282
Approach Reach Height (m)	2.90 ± 0.12	2.87 ± 0.12	0.434
Half-Meter Square Agility (s)	17.89 ± 1.01	18.09 ± 0.98	0.529
Standing Long Jump (m)	2.31 ± 0.23	2.28 ± 0.28	0.713
30 m Sprint (s)	4.62 ± 0.38	4.72 ± 0.39	0.417
1,500 m Run (s)	363.77 ± 35.43	377.79 ± 35.39	0.218

Statistically significant difference at *P* < 0.05.

### 2.3 Training program

Over the 12-week intervention period (2024 November 4 to 2025 January 26) at the men’s volleyball training facility in Huangshi, Hubei Province, China, both groups trained three times per week, each session lasting approximately 90 min and comprising warm-up, main training, and cool-down/stretching. All sessions were led by a professionally qualified coach to ensure movement standardization and safety. Attendance was recorded throughout, and only participants who completed at least 80% of all sessions were included in the final data analysis; those who failed to meet this threshold were excluded from analysis.

During the warm-up phase, participants in both groups followed an identical routine designed to fully activate the lower-limb musculature, enhance neuromuscular excitability, and prevent injury. This began with 5 min of jogging (treadmill optional), followed by dynamic stretching focused on lower-limb joint mobility and muscle extensibility. Dynamic stretches included the lunge with shoulder press and trunk rotation, side lunge squat, walking knee hugs, high-knee walks, and front–back leg swings. After dynamic stretching, unloaded plyometric activations—such as bodyweight squats, single-leg squats, in-place double-leg jumps, double-leg forward–backward hops, and lateral hops—were performed to further improve coordination and reaction speed. Total warm-up duration was kept under 15 min.

In the main training phase, due to the fundamental differences in training objectives between the two protocols, the mechanical loads applied to the two groups were not entirely equivalent. However, we strived to maintain comparable training density and neuromuscular stimulation levels. All sessions were supervised by the same certified strength and conditioning coach to ensure strict control over movement quality and training intensity. The control group followed a traditional lower-limb resistance protocol: barbell back squats (85% 1RM, six reps/set, three sets, 90 s rest), barbell half-squats (80% 1RM, 10 reps/set, three sets, 60 s rest), conventional deadlifts (≈80% maximal capacity, six reps/set, three sets, 60 s rest), weighted calf raises (40 kg, 12 reps/set, three sets, 60 s rest) and leg presses (1.2 × squat 1RM, six reps/set, three sets, 60 s rest). The experimental group performed a functional strength-training regimen integrating strength, balance, and power: single-leg lateral drop hops (bodyweight, 12 reps/set, three sets, 60 s rest), single-leg box jumps (bodyweight, 10 reps/set, three sets, 60 s rest), box squats with approach jumps (1RM load, six reps/set, three sets, each set immediately followed by two approach jumps; 90 s rest), single-leg Romanian deadlifts (10 kg, 12 reps/set, three sets, 60 s rest), forward loaded lunges (20 kg, 20 reps/set, three sets, 60 s rest) and reverse loaded walking lunges (20 kg, 20 reps/set, three sets, 60 s rest). All exercises were supervised by the same certified strength and conditioning coach to strictly control movement quality and training intensity (Table 2).

**TABLE 2:** Training program.

Exercise no.	Experimental group (functional training)	Control group (traditional resistance training)
1	Single-leg lateral drop jump (bodyweight, 12 reps/set, 3 sets, 60 s rest)	Barbell back squat (85% 1RM, 6 reps/set, 3 sets, 90 s rest)
2	Single-leg box jump (bodyweight, 10 reps/set, 3 sets, 60 s rest)	Barbell half squat (80% 1RM, 10 reps/set, 3 sets, 60 s rest)
3	Box squat + approach reach jump (1RM load, 6 reps/set, 3 sets, 90 s rest)	Conventional deadlift (∼80%, 6 reps/set, 3 sets, 60 s rest)
4	Single-leg Romanian deadlift (10 kg, 12 reps/set, 3 sets, 60 s rest)	Weighted calf raise (40 kg, 12 reps/set, 3 sets, 60 s rest)
5	Forward loaded lunge (20 kg, 20 reps/set, 3 sets, 60 s rest)	Leg press (1.2 × squat 1RM, 6 reps/set, 3 sets, 60 s rest)
6	Reverse loaded walking lunge (20 kg, 20 reps/set, 3 sets, 60 s rest)	—

For cool-down/stretching, both groups performed identical static stretches to promote muscle recovery, reduce delayed-onset muscle soreness, and minimize injury risk. Stretches included the seated forward bend, seated hamstring stretch, seated adductor stretch, hurdler’s stretch, and calf muscle stretch. Each stretch was held for 30 s, with total cool-down time limited to 10 min to ensure sufficient post-exercise recovery.

### 2.4 Evaluation indicators and methods

To ensure accuracy and reproducibility of test data, all assessments were conducted following standardized procedures using validated equipment. Testing took place at the men’s volleyball training facility in Huangshi, Hubei Province, with an electronic reach-height device, tape measure, stopwatch, and markers. To minimize external variability, all participants wore uniform athletic clothing and shoes, completed a standardized warm-up to activate neuromuscular readiness and reduce injury risk, and then performed each test at maximal effort. A team of trained physical-education instructors and research assistants administered and recorded all tests; each indicator was measured twice, with the best result used for analysis. Data were logged on standardized forms and cross-checked by two staff members. The specific tests and methods were as follows.

1. Standing Reach Height: Assesses lower-limb vertical explosive power. The subject stood within the testing zone with feet shoulder-width apart, swung the arms back, then jumped vertically with maximal effort to touch the electronic reach-height device with one hand. Both feet had to leave the ground simultaneously, and no preparatory step or forward movement was allowed. Two attempts were recorded; the highest value was used.
2. Approach Reach Height: Evaluates dynamic jump ability and approach-run power. The subject chose a comfortable approach distance, ran freely, then jumped with both feet and touched the reach-height device with one hand. The run-to-jump transition had to be smooth and continuous. Two trials were performed; the maximal height was recorded.
3. Half-Meter Square Agility Test: Measures agility and rapid change-of-direction ability. Six markers were arranged around the attack line and baseline of the volleyball court. Starting at marker 1, the subject ran the prescribed route, knocking down each marker by hand in sequence, and returned to marker 1. Timing stopped when marker one was last knocked down. If any marker was missed or the route was not followed, that segment was repeated.
4. Standing Long Jump: Assesses horizontal explosive power. The subject stood behind the take-off line with both feet, without stepping on the line or taking an approach. On cue, the subject swung the arms, jumped forward with both feet, and landed. The distance from the nearest heel to the take-off line was measured in centimeters. Two trials were conducted; the best distance was used.
5. 30 m Sprint: Tests short-distance acceleration and speed. The subject adopted a standing start behind the start line; the front foot could not cross the line. On the start signal, timing began and stopped when the torso crossed the 30 m finish line. Any false starts or rule violations invalidated the attempt, which was then repeated.
6. 1 500 m Endurance Run: Evaluates aerobic endurance and cardiovascular capacity. The subject ran three full laps plus 300 m on a standard 400 m track from a standing start. Timing began on the “go” command and stopped when the torso crossed the finish line. Continuous running was required; cutting corners invalidated the result.

### 2.5 Statistical methods

All data were analyzed using IBM SPSS Statistics 26.0. Prior to formal hypothesis testing, continuous variables were assessed for normality (Shapiro–Wilk test) and homogeneity of variance (Levene’s test) to verify the prerequisites for parametric analyses. The results of both tests showed that all variables had P-values greater than 0.05, indicating that the assumptions for parametric analyses were met. Therefore, all primary outcome measures were approximately normally distributed and suitable for parametric testing. For within-group comparisons, paired-samples t-tests (Paired Samples t-test) were applied to evaluate pre-to post-intervention changes in each group’s fitness indicators. For between-group comparisons, independent-samples t-tests (Independent Samples t-test) were used to compare post-intervention scores between the functional training and traditional training groups. Continuous variables are expressed as mean ± standard deviation (Mean ± SD), with the significance level set at α = 0.05; differences were considered statistically significant at P < 0.05.

## 3 Results and analysis

### 3.1 Comparison of lower-limb performance indicators before and after training in the control group

As shown in Table 3, after 12 weeks of traditional resistance training, the control group exhibited minimal overall changes in lower-limb performance indicators. Standing reach height increased from 2.85 ± 0.13 m to 2.90 ± 0.13 m (P = 0.231), and approach reach height from 2.90 ± 0.12 m to 2.94 ± 0.11 m (P = 0.279), neither of which reached statistical significance. However, the effect sizes for these two indicators were moderate (Cohen’s d = 0.38 and 0.35, respectively), suggesting a possible training-related trend despite the lack of statistical significance. The half-meter square agility test time decreased from 17.89 ± 1.01 s to 17.17 ± 0.98 s, with a statistically significant improvement (P = 0.028) and a moderate-to-large negative effect size (Cohen’s d = −0.72), indicating a beneficial effect of this training modality on agility. Standing long jump (d = 0.27), 30 m sprint (d = −0.06), and 1,500 m run (d = −0.21) showed only small, non-significant changes (all P > 0.05), with correspondingly small effect sizes. Overall, traditional resistance training appears limited in its capacity to improve lower-limb power, although it may have some potential in enhancing agility.

**TABLE 3:** Pre- and post-training lower-limb performance metrics in the control group.

Indicator	Pre-training (n = 20)	Post-training (n = 20)	Difference	Cohen’s d	P-value
Standing Reach Height (m)	2.85 ± 0.13	2.90 ± 0.13	0.05	0.38	0.231
Approach Reach Height (m)	2.90 ± 0.12	2.94 ± 0.11	0.04	0.35	0.279
Half-Meter Square Agility (s)	17.89 ± 1.01	17.17 ± 0.98	0.73	−0.72	0.028*
Standing Long Jump (m)	2.31 ± 0.23	2.37 ± 0.22	0.07	0.27	0.404
30 m Sprint (s)	4.62 ± 0.38	4.60 ± 0.27	0.02	−0.06	0.849
1,500 m Run (s)	363.77 ± 35.43	356.00 ± 37.64	7.77	−0.21	0.506

* Statistically significant difference at *P* < 0.05.

### 3.2 Comparison of lower-limb performance indicators before and after training in the experimental group

From the data in Table 4, after 12 weeks of functional training intervention, the experimental group showed positive changes across multiple measures. Standing reach height increased from 2.81 ± 0.10 m to 2.88 ± 0.09 m (P = 0.025), and approach reach height rose from 2.87 ± 0.12 m to 2.95 ± 0.11 m (P = 0.034), both reaching statistical significance. The effect sizes for these two variables were moderately large (Cohen’s d = 0.74 and 0.69, respectively), indicating that functional training effectively enhances lower-limb explosive power. Although the half-meter square agility time decreased from 18.09 ± 0.98 s to 17.50 ± 1.06 s, this change did not reach statistical significance (P = 0.075), it exhibited a moderate effect size (Cohen’s d = −0.58), suggesting a potential benefit in agility. Other measures—standing long jump (d = 0.21), 30 m sprint (d = −0.35), and 1,500 m run (d = −0.25)—also showed numerical improvements, but these were not statistically significant (all P > 0.05). Nevertheless, the corresponding small-to-moderate effect sizes suggest some training-related influence. Overall, functional training demonstrated a more pronounced and practically meaningful effect on improving lower-limb vertical explosive power, with additional potential benefits in agility.

**TABLE 4:** Pre- and post-training lower-limb performance metrics in the experimental group (functional training).

Indicator	Pre-training (n = 20)	Post-training (n = 20)	Difference	Cohen’s d	P-value
Standing Reach Height (m)	2.81 ± 0.10	2.88 ± 0.09	0.07	0.74	0.025*
Approach Reach Height (m)	2.87 ± 0.12	2.95 ± 0.11	0.08	0.69	0.034*
Half-Meter Square Agility (s)	18.09 ± 0.98	17.50 ± 1.06	0.59	−0.58	0.075
Standing Long Jump (m)	2.28 ± 0.28	2.34 ± 0.28	0.06	0.21	0.502
30 m Sprint (s)	4.72 ± 0.39	4.57 ± 0.47	0.15	−0.35	0.279
1,500 m Run (s)	377.79 ± 35.39	369.43 ± 32.83	8.36	−0.25	0.443

* Statistically significant difference at *P* < 0.05.

### 3.3 Between-group comparison of lower-limb performance indicators after training

As shown in Table 5, post-intervention comparisons between the two groups revealed no statistically significant differences in any fitness measure: standing reach height (P = 0.575), approach reach height (P = 0.775), half-meter square agility (P = 0.313), standing long jump (P = 0.708), 30 m sprint (P = 0.806), and 1,500 m run (P = 0.237), all P > 0.05. This indicates that, overall, functional training and traditional resistance training produced similar outcomes. However, Cohen’s d effect size analysis provided more details. Although no statistical significance was found, the experimental group showed advantages in standing reach height (d = −0.18) and standing long jump (d = −0.12), while the control group showed advantages in agility (d = 0.32) and endurance (1,500 m run, d = 0.38), indicating that traditional resistance training performed better in these areas. Overall, although no significant differences were observed between the two groups in most fitness measures, functional training still shows potential in certain specific abilities, especially in lower-limb explosive power, but larger sample sizes or longer intervention periods are needed to further verify these long-term effects.

**TABLE 5:** Post-training lower-limb performance metrics in the control and experimental groups.

Indicator	Control group (traditional resistance training, n = 20)	Experimental group (functional training, n = 20)	Cohen’s d	P-value
Standing Reach Height (m)	2.90 ± 0.13	2.88 ± 0.09	−0.18	0.575
Approach Reach Height (m)	2.94 ± 0.11	2.95 ± 0.11	0.09	0.775
Half-Meter Square Agility (s)	17.17 ± 0.98	17.50 ± 1.06	0.32	0.313
Standing Long Jump (m)	2.37 ± 0.22	2.34 ± 0.28	−0.12	0.708
30 m Sprint (s)	4.60 ± 0.27	4.57 ± 0.47	−0.08	0.806
1,500 m Run (s)	356.00 ± 37.64	369.43 ± 32.83	0.38	0.237

Statistically significant difference at *P* < 0.05.

## 4 Discussion

The present study demonstrates that functional training exerts a positive effect on lower-limb performance in adolescent male volleyball players, although the two training modalities yielded different improvements across specific measures. The experimental group (functional training) achieved significant gains in standing reach and approach reach (approximately +0.07–0.08 m, P < 0.05), whereas the control group (traditional training) exhibited smaller, non-significant improvements (approximately +0.04–0.05 m, P > 0.05). In the half-meter square agility test, the control group’s time decreased by ≈ 0.72 s (P < 0.05), while the experimental group improved by ≈ 0.59 s (P = 0.075), showing a favorable trend without reaching statistical significance. Both groups made modest, non-significant advances in standing long jump, 30 m sprint, and 1 500 m run: the experimental group’s standing long jump increased by ≈ 5 cm *versus* ≈6 cm in the control group; the 30 m sprint time decreased by 0.15 s *versus* 0.02 s, respectively; and both groups trimmed ≈ 5–8 s from their 1 500 m runs. Overall, functional training produced more pronounced gains in vertical explosive power, whereas traditional training yielded greater improvement in specific agility. These differing outcomes provide a foundation for further exploration of the mechanisms underlying functional *versus* traditional training.

Our findings that functional training significantly enhances vertical-jump metrics align with several domestic and international studies. One investigation in youth athletes reported that 8 weeks of functional training delivered greater improvements in 30 m sprint speed and vertical-jump height compared with traditional training (Bashir et al., 2022) Park et al. also found that 6 weeks of functional training in elite taekwondo athletes led to significant increases in standing long jump and Sargent vertical jump (Khazaei et al., 2023). The superior effect of functional training on explosive power may stem from its emphasis on multi-joint, dynamic movements, which improve neuromuscular recruitment efficiency and intermuscular coordination (Khazaei et al., 2023; Legerlotz et al., 2016). Functional protocols often incorporate rapid stretch-shortening cycle exercises (e.g., various jumps), akin to targeted plyometric drills, which facilitate jump performance in adolescent athletes (Xiao et al., 2021). A meta-analysis of youth volleyball players similarly concluded that power-focused training markedly enhances both vertical and horizontal jump performance and agility speed (Silva et al., 2019)。

Conversely, some research has questioned the jump-performance advantage of functional training. Keiner et al. reported that, in youth players, a traditional strength-training group achieved greater improvements in sprint speed and squat-jump height than a functional training group, noting that elastic-band and bodyweight-based drills may not provide sufficient overload to increase maximal strength in the short term (Bashir et al., 2022). Such discrepancies likely reflect differences in training content and load intensity: when “traditional” protocols include high-intensity resistance or specialized plyometric regimens, their direct stimulus on explosive power can exceed that of functional training, which primarily targets core stability and movement coordination.

In the current study, the control group’s conventional training—largely technical and lacking dedicated strength-enhancement components—may explain its limited jump-height gains, whereas the experimental group’s functional regimen, combining whole-body strength and jump drills, effectively activated lower-limb muscle synergies to drive significant vertical-jump increases. It is noteworthy that neither group showed marked improvement in 30 m sprint or 1 500 m endurance run. This finding is consistent with literature indicating that strength-dominant protocols—functional or traditional—without specific sprint or endurance sessions seldom yield significant short-term gains in linear speed or aerobic capacity (Haugen et al., 2019; Ozaki et al., 2013) The absence of 1 500 m run improvement was expected, as volleyball training rarely involves extended endurance work, making large aerobic adaptations unlikely over a few weeks (Bobula et al., 2024).

Overall, our jump and agility outcomes both mirror and diverge from published reports. The larger vertical-jump gains in the functional training group underscore its value for explosive power, yet the control group’s superior agility-test results were unexpected. Functional training—through its multi-directional, complex drills—should theoretically boost agility (Ft et al., 2021; Lee et al., 2023). In our design, however, the control group may have benefited from incidental reinforcement of change-of-direction movements aligned with the half-meter square test (reflecting the specificity principle), as our functional training plan emphasized strength training rather than agility-specific exercises and did not incorporate additional exercises for multidirectional movement and related qualities. Although the functional group enhanced core strength and balance control, its movement patterns did not perfectly match the agility-test demands, leading to smaller agility gains. A study even reported that resistance training did not alter speed or agility in female volleyball players (Arazi et al., 2018) These findings suggest that, while functional training develops global physical qualities, it must be combined with targeted agility drills to ensure effective transfer to specific competitive movements.

For adolescent athletes, functional training should focus on optimizing the integrated development of the locomotor system (Weiss et al., 2010) Grounded in the characteristics of holistic adolescent development, key principles have been proposed to foster the mutual promotion of skill acquisition and musculoskeletal strength and to guide mind–body synergy in youth functional training (Lloyd et al., 2015) Preliminary adolescent progressive functional-training frameworks have been established accordingly. Functional training offers benefits for enhancing athletic performance and injury prevention and for translating general fitness into sport-specific qualities, but the value of traditional conditioning cannot be dismissed (Latorre Román et al., 2015; Liao et al., 2021). Therefore, functional and traditional modalities should be used complementarily to promote high-level sport-specific capabilities.

### 4.1 Limitations

This study has several limitations. First, the sample size was relatively small and limited to a specific group of adolescent male volleyball players. The statistical power was 0.69, which is slightly below the commonly recommended threshold of 0.80, potentially indicating insufficient statistical efficiency and limiting the interpretability and generalizability of the findings. Second, the assessment battery was confined to lower-limb strength and fitness measures; we did not evaluate sport-specific technical performance (e.g., spike height, spike force) or other relevant attributes such as upper-limb strength, flexibility, and core stability. These omissions preclude a comprehensive understanding of functional training’s impact on all facets of athletic performance. Third, the study did not include post-intervention testing of participants’ maximal strength (e.g., 1RM), making it difficult to determine whether the intervention resulted in meaningful improvements in maximal strength. Finally, the experimental design lacked a passive control group, which prevents us from distinguishing between training effects and natural developmental changes in adolescents, thereby limiting the ability to establish clear causal inferences.

### 4.2 Future directions

Future studies should, in addition to the current fitness measures, incorporate tests closely aligned with volleyball performance—such as approach-jump spike height, spike velocity, lateral and change-of-direction speed, and in-game technical statistics—to assess the transfer of functional training to sport skills. Moreover, additional assessments (e.g., FMS functional movement screening, core-strength tests, lower-limb 1 RM) should be employed to capture a holistic profile of athlete development.

## 5 Conclusion

This study’s comparison of functional and traditional training in adolescent male volleyball players highlights that each modality has distinct advantages: functional training excels in enhancing explosive power and overall physical fitness, whereas traditional resistance training retains its edge in specific agility. These results—partly consistent and partly divergent from existing literature—reflect the context- and program-dependent nature of training adaptations. As a practical-transformation-oriented method, functional training offers unique value for improving explosive power, agility, and movement control in youth athletes but cannot wholly replace traditional resistance training for building absolute strength. Coaches should integrate both approaches, leveraging their respective strengths to maximize training outcomes. We look forward to more high-quality research in this field to refine youth athletic-conditioning protocols and provide robust scientific support for performance enhancement.

## Data Availability

The raw data supporting the conclusions of this article will be made available by the authors, without undue reservation.
